# Are attitudes toward music therapy associated with lower social anxiety in university students? The roles of emotion regulation and psychological flexibility

**DOI:** 10.3389/fpsyg.2026.1824427

**Published:** 2026-07-06

**Authors:** Pingting Li, Xiong Gao

**Affiliations:** 1 School of Art, Zhangjiajie College, Zhangjiajie, Hunan, China; 2 Department of Student Affairs, Zhangjiajie College, Zhangjiajie, Hunan, China

**Keywords:** attitudes toward music therapy, emotional regulation, psychological flexibility, social anxiety, university students

## Abstract

**Background:**

Social anxiety in university students is a mental health risk and is linked to various psychological resources. Emotional regulation and psychological flexibility are related to how students manage distress. This study explores how these factors are associated with the relationship between attitudes toward music therapy and social anxiety in students.

**Methods:**

This study collected questionnaire data from 689 university students in China through class-based convenience sampling. Established scales were used to measure social anxiety, emotion regulation, and psychological flexibility, while attitudes toward music therapy were assessed using a previously used seven-item Music Therapy Scale. Structural equation modeling was used to test the hypotheses.

**Results:**

Attitudes toward music therapy were negatively associated with social anxiety. Cognitive reappraisal (β = −0.147, *t* = 8.685, *p* < 0.001) and expressive suppression (β = −0.115, *t* = 7.641, *p* < 0.001) showed significant indirect associations in this relationship. Psychological flexibility moderated the relationship between attitudes toward music therapy and social anxiety (β = −0.176, *t* = 7.483, *p* < 0.001). Students with higher psychological flexibility showed a stronger negative link between attitudes toward music therapy and social anxiety compared to those with lower psychological flexibility.

**Conclusion:**

This study provides preliminary correlational evidence that university students’ attitudes toward music therapy are associated with social anxiety, emotion regulation, and psychological flexibility. The findings should not be interpreted as evidence for the effectiveness of music therapy intervention.

## 1 Introduction

Among students, social anxiety (SA) remains a frequent psychological concern. In the DSM-5, this condition refers to pronounced anxiety or fear arising in social settings that involve the possibility of being watched, judged, or closely examined by other people (American Psychiatric Association [APA], 2013). Students with SA often experience nervousness, shame, embarrassment, and avoidance in interpersonal or performance-related situations (Xu and Li, 2024). Among the core features of SA, fear of negative evaluation is particularly prominent, as affected individuals tend to worry excessively about being judged, rejected, or humiliated by others (Heimberg et al., 2014). At university, students face difficulties in their studies, changes in their social roles, and problems in their interpersonal relationships. These experiences are closely related to their psychological adjustment and quality of life. Academic performance and social interactions are also linked to students’ broader university experiences (Gong et al., 2025; Li and Zheng, 2025). Existing research among U.S. college students has reported that approximately 12% of students experience SA (Kraft et al., 2021). Although this estimate is not based on a Chinese sample, it still highlights the need for continued attention to SA and campus-based mental health support among university students.

In recent years, music therapy has attracted increasing attention as a non-pharmacological approach to supporting students’ emotional and mental health (Zhang et al., 2022; Li et al., 2026). In this context, students’ attitudes toward music therapy (ATMT) are important because they may be related to their willingness to engage with music-therapy-based support (Li et al., 2013). Music therapy (MT) refers to the professional, research-based use of music in clinical, educational, and everyday settings to support wellbeing, social connection, emotional experience, physical health, and spiritual growth (Haase, 2012). This study was not designed to assess students’ participation in an MT program. Its focus was on how university students perceive and evaluate music therapy, namely their ATMT. In recent years, music-based psychological support has been discussed in connection with emotional and mental health (Matokhniuk et al., 2021). Auditory elements such as melody, rhythmic patterns, and sound may relate to how individuals experience emotions, and they may also be connected with reduced emotional tension and anxiety (van Essen et al., 2025). Among students, positive ATMT may reflect greater openness to music-related emotional support and expression in stressful social situations (Qing et al., 2024). The emotion regulation process model (ERPM) (Gross, 1998) shows that individuals use different strategies to manage their emotions and adapt to their external circumstances and internal needs. Emotion regulation is closely related to SA, particularly because socially anxious individuals often interpret social situations through the lens of possible rejection or criticism (Dryman and Heimberg, 2018). In this context, fear of negative evaluation may make cognitive reappraisal (CR) especially relevant, as CR involves reinterpreting potentially threatening social cues in a more adaptive and balanced way (Gross and John, 2003; Goldin et al., 2009). Unlike this pattern, expressive suppression (ES) involves restraining emotional displays only after the emotional experience has already occurred (Gross and John, 2003). In social situations marked by stress, this strategy is commonly considered less constructive or less suitable (Dryman and Heimberg, 2018; Kivity et al., 2021; McBride et al., 2022; Mathijs et al., 2024). Psychological flexibility is an important psychological resource and is closely related to individual pressure and anxiety (Guerrini Usubini et al., 2021). People with higher psychological flexibility often show greater adaptability in response to internal and external demands. It may also be related to the management of SA (Pakenham et al., 2020; Figueiredo et al., 2024).

Studies have begun to focus on the connection between music-related psychological support, emotion regulation, psychological flexibility, and SA (McBride et al., 2022; Arizona et al., 2023; Figueiredo et al., 2024; Qing et al., 2024). However, there are still some gaps in the literature. Few studies have examined how ATMT may be linked to specific emotion regulation strategies, such as CR and ES, in relation to students’ SA. Research has also not clearly addressed how psychological flexibility may be involved in this relationship. Drawing on both ERPM and ACT, this study considers the association between ATMT and SA in a sample of university students. This study further considers two related questions: whether CR and ES may account for part of this relationship, and whether psychological flexibility may be linked to variation in the strength or pattern of this relationship. Using this multidimensional model as a basis, the study further explores psychological patterns related to SA in student populations. These findings may help enrich academic understanding and inform mental health support practices in university contexts.

## 2 Theoretical framework and hypotheses

### 2.1 Theoretical foundation

ERPM was proposed by Gross (1998). It shows how to use different strategies to control one’s emotions. The process influences people’s mental health and behavior. The model shows that emotional regulation includes CR and ES. These strategies are associated with emotional experiences and psychological reactions through a variety of mechanisms (Gross, 2013). Previous studies have linked music-related psychological support to emotion regulation strategies (Peters et al., 2024). In the present study, the focus is on ATMT, rather than participation in formal MT intervention. ATMT may show a positive association with CR and a negative association with ES (Yue, 2025). Music-related emotional experiences may also be linked to the shaping and rebalancing of individuals’ emotional responses (van Essen et al., 2025). These emotional processes may be related to lower levels of SA (McBride et al., 2022).

ACT was developed by Hayes et al. (2011). It is a psychological approach that focuses on acceptance and commitment. It emphasizes openness to negative emotions and thoughts rather than avoidance or excessive control. In ACT, psychological flexibility involves accepting emotions rather than resisting them. When people accept painful emotions, they may show less reliance on suppression or avoidance. This is related to greater adaptability in facing life challenges (Hayes et al., 2011). People learn to face their negative feelings and accept them, and therefore adapt more easily. They can behave in accordance with their values and goals, regardless of emotions (Hayes et al., 2011). For university students with stronger psychological flexibility, ATMT may correspond to a greater willingness to engage with emotions elicited by music and to connect these experiences with emotional regulation strategies (Juncos et al., 2017). Existing studies indicate that higher psychological flexibility is associated with less rigid responses to anxiety-related emotional experiences and lower SA (Figueiredo et al., 2024). Those who are more psychologically flexible may also show better adaptation to stress and greater openness to their environment (Guerrini Usubini et al., 2021; Figueiredo et al., 2024).

### 2.2 Hypotheses

#### 2.2.1 Attitudes toward music therapy and social anxiety

Within the ERPM framework, the auditory features of music, including rhythmic patterns, melodic structure, and harmonic organization, may relate to how individuals experience emotions and may be associated with a calmer and more balanced emotional state (van Essen et al., 2025). Therefore, students with more positive ATMT may also show greater openness to music-related emotional support in stressful social situations.

Although direct evidence on the association between ATMT and SA remains limited, related studies provide indirect support for this possible relationship. Studies on ATMT suggest that positive ATMT are linked to greater willingness to engage with MT or music-related psychological support (Zhang and Lay, 2024; Carney et al., 2025). In addition, existing research has shown a negative correlation between music-related experiences and SA (Arizona et al., 2023; Qing et al., 2024; de Witte et al., 2025). Arizona et al. (2023) noted in their study that MT, as an emerging intervention field, shows potential in promoting social and emotional health. The study highlights that using ethnic music in MT can provide a more culturally suitable support option for people with SA in public health settings. A study conducted by the University of Qing et al. (2024), among others, involving 902 students from Beijing examined the relationship between participatory musical activities and SA. The results showed that participatory music activities are negatively linked to SA. This finding broadens the understanding of how music participation is linked to social and emotional experiences in university students. de Witte et al. (2025) carried out a systematic review paired with a multilevel meta-analysis to integrate research on MT and anxiety across various settings. They noted that MT is a personalized approach delivered by professional therapists and is linked to emotional expression and regulation through personalized music experiences within the therapeutic relationship. These studies provide a useful basis for considering why students’ positive ATMT may be related to lower SA.

#### 2.2.2 Mediating role of emotional regulation

Emotional regulation refers to the ways individuals adjust their emotional experiences and emotional expression in response to situational demands and personal needs (Gross, 1998). Researchers have paid particular attention to CR and ES within the broader field of emotion regulation strategies. CR is often regarded as an adaptive emotional regulation strategy because it helps individuals reconsider the meaning of an emotion-related situation before their emotional response is fully formed. This strategy is often associated with lower negative affect and more favorable psychological adjustment (Gross, 1998). By contrast, ES is typically regarded as a less adaptive strategy because it operates at a later stage of the emotion-generative process and focuses on inhibiting outward emotional expression after the emotion has already arisen (Gross, 2002). Although ES may be related to more controlled visible emotional reactions in the short term, persistent reliance on this strategy may be associated with heightened internal tension and less favorable mental health outcomes, including greater emotional distress and poorer psychological functioning (Chen et al., 2025).

Existing research has linked MT and music-related activities to emotion regulation (Zhang et al., 2022; Peters et al., 2024; Tan et al., 2024; Gaebel et al., 2025; Lund and Drago, 2025; Yue, 2025). Peters et al. (2024) discovered that music interventions, including listening, playing, and creating, were strongly linked to better emotional regulation. The longer the intervention lasted, the stronger this connection was. Yue (2025) studied 240 children aged 8–10. The study found that emotional expression and reflection in MT were closely related to more use of CR. It also showed a trend of less ES. Although these studies mainly focused on music interventions or music activities rather than ATMT, they suggest that music-related emotional experiences may be associated with how individuals understand and regulate their feelings. Building on this evidence, more positive ATMT among university students may correspond to greater openness to music-related emotional experiences, which may be associated with more frequent use of CR and less reliance on ES.

Research shows a strong connection between emotional regulation and SA (Kivity et al., 2021; McBride et al., 2022; Mathijs et al., 2024). McBride, Bates (McBride et al., 2022) studied individuals with SA disorder seeking treatment and university students with high and low SA. The study found that emotional regulation strategies, particularly CR and ES, were directly related to the expression of SA. Mathijs et al. (2024) conducted a study with 278 Swiss adolescents and further showed that emotional dysregulation and emotional suppression are mediating mechanisms between overprotective parenting and SA. They emphasized that adolescents with insufficient emotional regulation skills are more likely to experience anxiety symptoms in social situations. Kivity et al. (2021) studied individuals with SA disorder undergoing cognitive behavioral therapy and found that a decrease in the frequency of ES and a reduction in neural responsiveness to social threat cues were closely related to symptom relief. This indicates that emotional regulation skills are closely related to SA. Emotional regulation may mediate the relationship between ATMT and SA.

#### 2.2.3 Moderating role of psychological flexibility

Psychological flexibility is the ability to adjust to changes in the environment. It means adapting thoughts and behaviors and choosing appropriate ways to handle stress or challenges (Hayes et al., 2011). This idea comes from ACT. It stresses that individuals should not allow negative thoughts or emotions to control them. Instead, they should accept and coexist with these thoughts and emotions. In order to achieve a meaningful life goal, they should remain open and adapt their behavior (Hayes et al., 2011). In the present study, psychological flexibility may be relevant to the relationship between ATMT and SA. Students with higher psychological flexibility may show greater receptiveness to music-related emotional experiences and may manage mood changes in a more flexible manner (Juncos et al., 2017). Some studies have reported a negative association between MT and anxiety under specific conditions (Lu et al., 2021). Existing studies also indicate that greater psychological flexibility is related to less rigid responses to anxiety-related emotional reactions and lower SA (Figueiredo et al., 2024). People with high psychological flexibility may be more capable of regulating their emotional responses (Pakenham et al., 2020). They usually adapt better to change and are more able to deal with stress (Guerrini Usubini et al., 2021). Therefore, students with higher psychological flexibility may show a stronger negative association between ATMT and SA. In contrast, students with lower psychological flexibility may respond to emotional experiences in a more avoidant or suppressive way, which may weaken the association between ATMT and SA. Psychological flexibility may therefore moderate the relationship between ATMT and SA.

**FIGURE 1:**
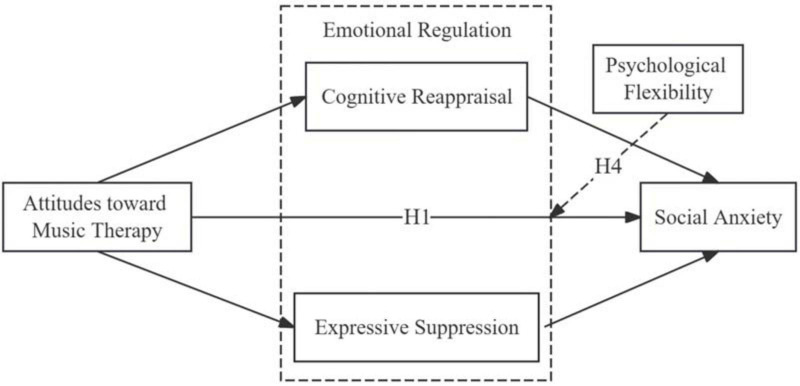
Research hypothesis model.

Using ERPM and ACT as theoretical frameworks, this study focuses on how ATMT is related to SA in university students. Emotional regulation is considered a mediator. Psychological flexibility is considered a moderator. This model may provide a clearer understanding of SA in university students. It may provide preliminary evidence for mental health education and for future research on professionally delivered music therapy. The proposed research model is illustrated in Figure 1. Drawing from this framework, we put forward the following hypotheses.

*H1*: ATMT is negatively correlated with SA in university students.

*H2*: CR mediates the relationship between ATMT and SA in university students.

*H3*: ES mediates the relationship between ATMT and SA in university students.

*H4*: Psychological flexibility moderates the relationship between ATMT and SA in university students.

## 3 Research methods

### 3.1 Sample and data collection procedure

Using a cross-sectional approach, this study focused on how ATMT was related to social anxiety. From September to November 2025, participants completed the survey on the Wenjuanxing online system,^1^ and we used these responses for the analysis. The study sample came from a range of universities located in different parts of China. A convenience sampling strategy was used, with participants recruited through class-based recruitment. During class meetings, advisors briefly introduced the survey and explained its purpose and relevance. After students indicated their willingness to participate, we arranged the survey administration in a unified way and collected their responses during a limited period. Students took part in the study voluntarily after providing informed consent, and participation was not compulsory. The sample covered students from different universities, which may provide broader participant variation. Even so, this sampling approach does not fully guarantee representativeness. All responses on the Wenjuanxing platform were anonymous and did not contain any personally identifiable information.

According to Kline (2018), a minimum of 10 participants per item was recommended. As the questionnaire included 42 items, the minimum required effective sample size was 420. Assuming a possible attrition rate of 20%, the target recruitment size was estimated at 525 participants (420/0.80 = 525). A total of 700 students agreed to participate and submitted the questionnaire during the arranged survey period. Because the survey was administered in a coordinated setting after informed consent had been obtained, a high completion rate was achieved. After cleaning the data, 11 questionnaires were removed. Eleven questionnaires were excluded because they were incomplete or showed extreme response patterns. Thus, 689 valid questionnaires were retained for analysis.

The demographic data are shown in Table 1. There were 335 males (48.6%) and 354 females (51.4%). Most participants were between 18 and 20 years old, with 540 participants (78.4%). The majority were first-year students, totaling 414 participants (60.1%). We also conducted independent sample *t*-tests and one-way ANOVA on key study variables. The aim was to examine whether SA differed by gender, grade, and age and to consider potential differences in SA across demographic factors. The results showed no significant differences in SA based on gender (*p* > 0.05), grade (*p* > 0.05), or age (*p* > 0.05). This sample provides a good basis for examining students’ SA.

**TABLE 1:** Demographic characteristics of participants.

Variable	Number	Percentage (%)	SA *p*-value
Gender			0.814
Male	335	48.6	
Female	354	51.4	
Age	540	78.4	0.073
18–20 years			
21–22 years	136	19.7	
23 years and above	13	1.9	
Grade	414	60.1	0.416
Freshman			
Sophomore	126	18.3	
Junior	84	12.2	
Senior	65	9.4	

### 3.2 Measurement tools

#### 3.2.1 Attitudes toward music therapy

ATMT was measured using the Music Therapy Scale (MTS) created by Liu (2024). However, this scale should not be regarded as a fully standardized measure of music therapy knowledge or participation in formal music therapy. Therefore, the findings related to this scale should be interpreted as reflecting students’ self-reported attitudes toward music therapy. The scale has 7 questions. In the present study, the scale was interpreted as a measure of students’ beliefs, perceived value, and openness toward music therapy, rather than as a measure of exposure to or outcomes of formal music therapy. A sample item is “I believe that music therapy may be helpful for emotional health.” It uses a 5-point Likert scale for scoring. A higher score indicates more positive ATMT. This scale had good internal consistency. The Cronbach’s α was 0.925 in this study. It should be noted that this scale was used to assess students’ attitudes, beliefs, and perceived value regarding MT, rather than their participation in a formal MT intervention.

#### 3.2.2 Social anxiety

SA was measured using the Social Anxiety Scales (SAS) developed by Leary (1983) and adapted by Xu and Li (2024). The scale has 15 items. One of them is “I often feel nervous at informal gatherings.” It uses a 5-point Likert scale for scoring. The higher the scores, the more severe the SA symptoms. In this study, the Cronbach’s α for the SA scale was 0.945. This shows it has good reliability.

#### 3.2.3 Emotion regulation

Emotional regulation was measured using the Emotion Regulation Questionnaire (ERQ). It was developed by Gross and John (2003) and adapted by Sheng et al. (2024). The scale has two dimensions: CR and ES. It includes a total of 10 items. The CR dimension has 6 items. One example is “I control my emotions by changing how I think about the current situation.” The ES dimension has 4 items. One example is “I hide my emotions.” The scale uses a 5-point Likert scale for scoring. In this study, the Cronbach’s α for the CR dimension was 0.916. For the ES dimension, it was 0.862. Both values show high reliability.

#### 3.2.4 Psychological flexibility

Psychological flexibility was measured using the Acceptance and Action Questionnaire-2nd Edition (AAQ-II). It was developed by Bond et al. (2011) and revised by Li et al. (2022). The scale has 7 items. One of them is “I am afraid of my emotions.” The scale uses a 5-point Likert scale. All items are reverse-scored. A higher score indicates higher psychological flexibility. In this study, the Cronbach’s α for the AAQ-II was 0.921. This shows good reliability.

This study used the translation-back-translation method. It helped check the accuracy of the language and the cultural fit of the questionnaire (Brislin, 1969). Two independent researchers did the initial translation. Among the experts, one had a background in educational psychology and demonstrated fluency in both English and Chinese. The other expert was a linguist with more knowledge of Chinese. The experts each prepared their own Chinese translation of the original English questionnaire. Later, another bilingual expert, who was not part of the first translation, translated it again. They compared it with the original version. They identified any biases or unclear sections. We made changes to the parts with differences. This helped ensure the content matched the original and was clear. To assess whether the questionnaire fit the Chinese cultural context, we invited 20 Chinese university students to take part in a preliminary test. They gave feedback on the questionnaire. They talked about how clear, natural, and suitable it was for the culture. We used their feedback to correct unclear or awkward items. This resulted in the final Chinese version. This process made sure the questionnaire was correct in language. It also fit the culture of the target group.

### 3.3 Statistical analysis

This study used AMOS 28 software to run confirmatory factor analysis (CFA). The goal is to check if the model is structurally valid. We also want to see if each variable is measured correctly. The data analysis was performed using PLS-SEM on SmartPLS 4.0. The aim was to explore the relationships between ATMT, emotional regulation (CR and ES), SA, and psychological flexibility. The research model has 5 latent variables and 42 measurement items. Data were obtained from 689 valid questionnaires. Given that the model included five latent variables and 42 measurement items, PLS-SEM was used to test the proposed structural relationships. This hybrid approach was adopted because the two methods served different but complementary purposes: AMOS was used to confirm the adequacy of the measurement model, whereas SmartPLS was used to test the structural relationships in the proposed model. Given that the research model included five latent variables and 42 measurement items, and was based on 689 valid questionnaires, the use of PLS-SEM was considered appropriate for analyzing the relatively complex model.

## 4 Results

### 4.1 Descriptive statistics and correlation analysis

Table 2 shows the descriptive statistics and correlation of the most important variables in this study. Means (M), standard deviations (SD), skewness (SK), and kurtosis (Kur) were reported. These values help to understand the concentration trends and distribution of a variable. This indicates that both the vector values and the principal values of all variables are within acceptable ranges, and that the data as a whole corresponds to a normal distribution.

**TABLE 2:** Descriptive statistics and correlation results analysis.

Constructs	*N*	*M* ± *SD*	*SK*	*Kur*	ATMT	CR	ES	PF	SA
ATMT	689	3.003 ± 1.179	0.013	−1.156	1	1	1	1	1
CR	689	3.006 ± 1.195	−0.040	−1.162	0.489***				
ES	689	2.989 ± 1.195	0.053	−1.153	−0.322***	−0.206***			
PF	689	2.976 ± 1.182	0.028	−1.159	0.359***	0.175***	−0.113***		
SA	689	3.020 ± 1.062	−0.136	−0.995	−0.542***	−0.508***	0.505***	−0.378***	

^***^p < 0.001.

The correlation analysis in Table 2 shows that ATMT is positively linked to CR (*r* = 0.489, *p* < 0.001) and psychological flexibility (*r* = 0.359, *p* < 0.001). It shows a significant negative correlation with SA (*r* = −0.542, *p* < 0.001) and ES (*r* = −0.322, *p* < 0.001). CR shows a significant positive correlation with psychological flexibility (*r* = 0.175, *p* < 0.001). It shows a significant negative correlation with SA (*r* = −0.508, *p* < 0.001). ES shows a significant negative correlation with psychological flexibility (*r* = −0.113, *p* < 0.001). It shows a significant positive correlation with SA (*r* = 0.505, *p* < 0.001). Psychological flexibility shows a significant negative correlation with SA (*r* = −0.378, *p* < 0.001).

### 4.2 Common method bias (CMB)

This study tested the issue of CMB using Harman’s single-factor modeling method. The results showed that there were 5 factors with eigenvalues greater than 1. The first factor accounted for 36.148% of the total variance. This did not reach the 40% threshold (Podsakoff et al., 2003). This suggests that the issue of CMB is relatively small.

We further used the unmeasured latent method factor approach to assess potential common method bias. We added an unmeasured methodology to the measurement model. The latent factor was connected to all observed variables. Then, we compared the goodness-of-fit between the model containing the method factor and the baseline model without the factor (Podsakoff et al., 2003). The results showed that the chi-square value for the baseline model was χ^2^ = 738.184 (*df* = 692), while the chi-square value for the model with the latent method factor was χ^2^ = 738.170 (*df* = 691). The chi-square difference between the two models was 0.014 (*df* = 1), and the difference was not significant (*p* > 0.05). The results imply that CMB is not a major factor affecting this study.

### 4.3 Confirmatory factor analysis

This study applied CFA to evaluate the adequacy of the measurement model. We determined whether the factor structure of a questionnaire corresponds to theoretical expectations in order to verify its validity in measuring potential variables. The results of the study are shown in Table 3.

**TABLE 3:** Model fit indices.

Fit index	Reference value	Final model
CMIN/DF	< 5	1.067
RMSEA	< 0.05	0.010
GFI	> 0.9	0.949
AGFI	> 0.9	0.943
CFI	> 0.9	0.997
IFI	> 0.9	0.997
TLI	> 0.9	0.997

### 4.4 Measurement model

This study tested the reliability and validity of the measurement model, based on Hair et al. (2021). Indicator reliability was assessed using factor loadings, and internal consistency reliability was examined using composite reliability. In general, factor loadings of 0.70 or higher are recommended (Hair et al., 2021). In the present study, a small number of indicators, including SA14 (0.671), were slightly below this recommended threshold. However, these items were retained because their loadings were still within an acceptable range, and the overall composite reliability and average variance extracted (AVE) of their corresponding constructs remained satisfactory. In particular, the AVE for the SA construct was 0.568, exceeding the recommended cutoff of 0.50, which suggests adequate convergent validity at the construct level. In addition, item retention was not determined solely by a rigid loading threshold. Theoretical relevance and content coverage were also taken into account, as deleting these items could have narrowed the conceptual representation of the construct. As shown in Table 4, all constructs demonstrated acceptable composite reliability values above 0.70, indicating good internal consistency (Hair et al., 2021).

**TABLE 4:** Reliability and validity of the model.

Constructs	Items	Loadings	Cronbach’s α	Composite reliability	AVE
CR	CR1	0.867	0.916	0.935	0.704
	CR2	0.849			
	CR3	0.841			
	CR4	0.825			
	CR5	0.832			
	CR6	0.820			
ES	ES1	0.883	0.862	0.906	0.707
	ES2	0.840			
	ES3	0.825			
	ES4	0.813			
ATMT	ATMT1	0.866	0.925	0.939	0.689
	ATMT2	0.864			
	ATMT3	0.837			
	ATMT4	0.812			
	ATMT5	0.840			
	ATMT6	0.795			
	ATMT7	0.794			
PF	PF1	0.847	0.921	0.937	0.679
	PF2	0.845			
	PF3	0.847			
	PF4	0.841			
	PF5	0.803			
	PF6	0.814			
	PF7	0.766			
SA	SA1	0.808	0.945	0.952	0.568
	SA2	0.780			
	SA3	0.802			
	SA4	0.782			
	SA5	0.783			
	SA6	0.744			
	SA7	0.782			
	SA8	0.764			
	SA9	0.760			
	SA10	0.754			
	SA11	0.735			
	SA12	0.717			
	SA13	0.708			
	SA14	0.671			
	SA15	0.704			

CR, composite reliability; AVE, average variance extracted.

For validity assessment, this study focused on testing discriminant validity. Discriminant validity was tested using both the HTMT and the Fornell-Larcker criterion. The results show that all HTMT values (Table 5) are below 0.90. The square roots of the AVE for each construct are higher than their correlations with other constructs (Table 6), which meets the Fornell-Larcker criterion. In conclusion, the measurement model in this study meets the reliability and validity criteria set by Hair et al. (2021).

**TABLE 5:** HTMT standards.

Constructs	CR	ES	ATMT	PF	SA
CR					
ES	0.232				
ATMT	0.531	0.361			
PF	0.190	0.127	0.390		
SA	0.545	0.560	0.580	0.405	

**TABLE 6:** Fornell-Larcker standards.

Constructs	CR	ES	ATMT	PF	SA
CR	***0.839***				
ES	−0.207	***0.841***			
ATMT	0.490	−0.323	***0.830***		
PF	0.177	−0.114	0.360	***0.824***	
SA	−0.509	0.507	−0.544	−0.380	***0.754***

The square root of AVE values is displayed in bold and italicized font on the diagonal in the table.

### 4.5 Structural model

#### 4.5.1 Collinearity test

Empirical standards suggest that the variance inflation factor (VIF) should be less than 3.3 (Hair et al., 2021). The results (Table 7) show that the VIF values for all variables in this study range from 1.000 to 1.561. This means the model does not have significant multicollinearity.

**TABLE 7:** Collinearity test.

Constructs	CR	ES	ATMT	PF	SA
CR					1.322
ES					1.121
ATMT	1.000	1.000			1.561
PF					1.149
SA					

#### 4.5.2 Path hypotheses

**FIGURE 2:**
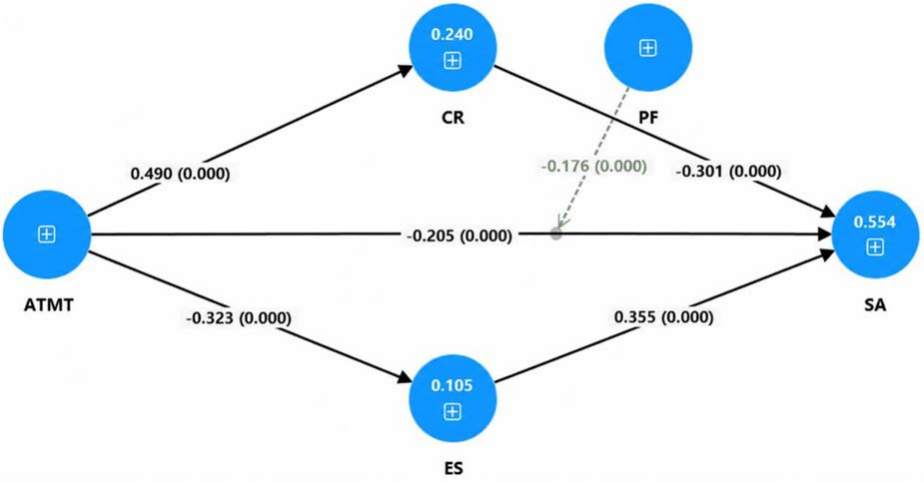
Path diagram after bootstrapping.

In the structural model test, this study used PLS-SEM. It estimated the path coefficients and their significance with 5,000 bootstrap resamples. The main results are shown in Table 8 and Figure 2. The analysis shows a significant negative association between ATMT and SA (β = −0.205, *t* = 6.499, *p* < 0.001).

**TABLE 8:** Mediating role of CR and ES in the relationship between ATMT and SA (*n* = 689).

Path	β	*t*	2.5%	97.5%	*p*	Result
CR ulSA	−0.301	10.395	−0.358	−0.244	< 0.001	Support
ES poSA	0.355	13.063	0.303	0.407	< 0.001	Support
ATMT → CR	0.490	16.411	0.431	0.550	< 0.001	Support
ATMT → ES	−0.323	9.707	−0.391	−0.260	< 0.001	Support
ATMT → SA	−0.205	6.499	−0.266	−0.143	< 0.001	Support
PF poSA	−0.213	7.432	−0.270	−0.159	< 0.001	Support

#### 4.5.3 Coefficient of determination

This study used the *R*^2^-value and *Q*^2^-value of the endogenous variables. These values help to determine the explanatory power and predictive relevance of a model (Hair et al., 2021). The results show that the R^2^ for SA is 0.554 (Table 9). This indicates that the model explained 55.4% of the variance in SA. All Q^2^ values are greater than zero. This suggests that the model has good predictive relevance.

**TABLE 9:** Explanatory power and predictive relevance.

Constructs	*R* ^2^	*Q* ^2^	Model fit
CR	0.240	0.237	SRMR: 0.031
ES	0.105	0.101	NFI: 0.941
SA	0.554	0.352	

R^2^ represents the explanatory power of the model; Q^2^ represents the predictive relevance of the model; NFI, normed fit index; SRMR, standardized root means square residual.

### 4.6 Mediation analysis

This study tested the mediating role of emotional regulation using the bootstrapping method (Nitzl et al., 2016). It calculated the indirect and direct effects using 5,000 subsamples. It tested if the mediation effect was significant and what type it was. The analysis results show that both ES (β = −0.115, *t* = 7.641, *p* < 0.001) and CR (β = −0.147, *t* = 8.685, *p* < 0.001) significantly mediate the relationship between ATMT and SA (Table 10).

**TABLE 10:** Mediation effect analysis.

Path	Indirect effect	*t*	*p*	Direct effect	*t*	*p*	Mediation type
ATMT → ES → SA	−0.115	7.641	<0.001	−0.205	6.499	<0.001	CPM
ATMT → CR → SA	−0.147	8.685	<0.001	−0.205	6.499	<0.001	CPM

CPM, complementary partial mediation.

### 4.7 Moderation analysis

This study used a two-stage method to examine the role of psychological flexibility in the relationship between ATMT and SA. The two-stage method is often used in moderation effect analysis. It is seen as more accurate than methods such as the product indicator method and the orthogonal method (Henseler and Chin, 2010; Becker et al., 2018). Before adding the interaction term, the R^2^ for SA was 0.485. This means that the model yields 48.5% of the variance. After including the interaction term between ATMT and psychological flexibility, the R^2^ value increased to 0.554. This shows a 6.9% improvement in the model’s ability to explain the variance. This means that psychological flexibility makes the model explain SA better. Further analysis showed that the interaction term was significant (β = −0.176, *t* = 7.483, *p* < 0.001). This suggests that psychological flexibility was associated with variation in the strength of the relationship between ATMT and SA. Details are shown in Table 11.

**TABLE 11:** Moderation analysis.

Path	β	*SE*	*t*	Bootstrap 95% CI		*p*
				LLCI	ULCI	
PF × ATMT → SA	−0.176	0.024	7.483	−0.222	−0.131	< 0.001

**FIGURE 3:**
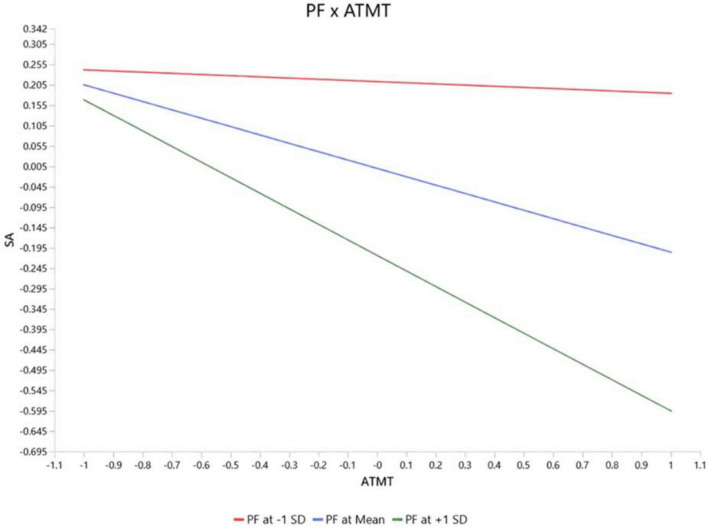
Simple slope analysis plot.

The results of the simple slope analysis (Figure 3) show that psychological flexibility was related to the strength of the association between ATMT and SA. The simple slope analysis showed that the negative association between ATMT and SA was weaker when PF was low and stronger when PF was high. This pattern suggests that students with higher PF may show a more pronounced negative association between ATMT and SA.

## 5 Discussion

The results showed that 55.4% of the variation in university students’ SA could be statistically accounted for by the proposed model (*R*^2^ = 0.554). This indicates that attitudes toward music therapy, emotion regulation, and psychological flexibility, taken together, were substantially associated with social anxiety in the present sample. This result also supports the value of the proposed framework for understanding social anxiety in university students. In addition, the *t*-test and ANOVA results showed no significant differences in social anxiety across gender, age, and grade groups. Although these were non-significant findings, they remain meaningful, as they suggest that the associations identified in the framework were relatively consistent across different student subgroups in the present sample.

This study found a significant negative correlation between ATMT and SA, confirming H1. Because the present study measured ATMT rather than participation in formal MT, this finding should be understood as evidence that students with more positive ATMT tended to report lower SA. Existing studies on music-related psychological support provide an indirect basis for understanding this relationship. For example, Arizona et al. (2023) suggested that MT is relevant to social and emotional health, while other studies have shown that music is associated with physiological stress indicators, such as heart rate and muscle tension (Song et al., 2024; Saskovets et al., 2025). These findings suggest that music-related experiences may be linked to emotional relaxation and lower tension in social situations (de Witte et al., 2025). In addition, auditory stimuli may be related to students’ attention shifting from anxiety-provoking social situations to more pleasant stimuli, which may correspond to lower SA symptoms (de Witte et al., 2020). Music activities, such as choir participation or group MT, are also linked to social connection, self-efficacy, and social support among university students (Qing et al., 2024). Based on this evidence, students with more positive ATMT may be more open to music-related emotional support and may hold more positive expectations about the value of such support. This may partly explain why ATMT was negatively associated with SA in the present sample. This finding may be especially meaningful because 60.1% of the sample consisted of first-year students. The transition to university is often accompanied by changes in academic demands, interpersonal relationships, living arrangements, and role expectations (Maymon and Hall, 2021). These transition-related stressors may be closely associated with social anxiety (Bloomfield et al., 2024). In this context, more positive ATMT may reflect greater openness to non-stigmatizing and emotionally safe forms of support during the transition period, which may be related to lower emotional tension, better adjustment to unfamiliar social environments, and a stronger sense of connection (Yang et al., 2025; Li et al., 2026).

Emotional regulation helps explain the link between ATMT and SA in university students. This supports H2 and H3. The ERPM (Gross, 2002) indicates that individuals may rely on emotional regulation strategies to organize their emotional experiences, and this process may relate to how they respond to external contexts and internal psychological needs. In this study, more favorable ATMT was positively related to CR and negatively related to ES. This pattern suggests that students with more positive ATMT may be more open to music-related emotional experiences and may show more adaptive emotion regulation patterns. Previous studies have also linked MT and music-related activities to relaxation, emotional expression, and emotion regulation (Yue, 2025). Students with more positive ATMT may be more likely to use CR to reinterpret social situations in a more balanced way, which is related to lower anxiety (Gaebel et al., 2025). In contrast, lower reliance on ES may be relevant because ES is often associated with greater internal tension and higher SA. Neuroscientific evidence also indicates that responses to music may correspond to activity in neural systems related to emotion processing, including the prefrontal cortex and amygdala (Ochsner and Gross, 2005; Peters et al., 2024). The prefrontal cortex is closely related to CR, whereas the amygdala is associated with anxiety-related responses (Ochsner and Gross, 2005). From this perspective, music-related emotional experiences may provide an indirect basis for understanding why ATMT was linked to CR, ES, and SA in the present study (Noda and Noda, 2025).

Psychological flexibility moderates the relationship between ATMT and SA among university students. University students with higher psychological flexibility showed a stronger negative link between ATMT and SA. For university students with lower psychological flexibility, this link is weaker. H4 is supported. One possible way to understand this moderating pattern is to consider psychological flexibility as the capacity to accept internal experiences without excessive avoidance and to deal with distress with greater adaptability (Lu et al., 2021). In the context of ATMT, students with higher psychological flexibility may be more open to music-related emotional experiences and more inclined to integrate these experiences into their ongoing emotion regulation process (Juncos et al., 2017; Guerrini Usubini et al., 2021; Figueiredo et al., 2024). In contrast, when psychological flexibility is lower, students may respond to distressing emotions in more avoidant, suppressive, or inflexible ways. Under such conditions, the association between positive ATMT and lower SA may become weaker (Pakenham et al., 2020). From this perspective, low psychological flexibility may be viewed as a psychological condition associated with a weaker link between ATMT and lower SA.

The present findings may also be better understood within the cultural context of Chinese university students. In Chinese cultural settings, values such as social harmony, emotional restraint, and sensitivity to interpersonal evaluation may shape how students respond to distress in social situations (Zeng and Zhu, 2021). Under these cultural expectations, students may be more likely to regulate negative emotions through expressive suppression in order to avoid interpersonal conflict or disrupting group harmony (Wei et al., 2013; Cui et al., 2022). At the same time, academic pressure, peer comparison, and family expectations may be related to greater concern about performance and evaluation, which may be relevant to anxiety in university settings (Zheng et al., 2023; Zhou et al., 2025). Against this background, ATMT may be particularly meaningful because positive ATMT may reflect greater openness to indirect, non-confrontational, and emotionally safe forms of expression and regulation (Zhang et al., 2022). In addition, culturally suitable support and the use of familiar or culturally resonant music materials, including ethnic music, may be related to students’ sense of acceptance, emotional engagement, and openness to music-related psychological support (Arizona et al., 2023). Therefore, the association between ATMT, emotion regulation, and SA may be partly shaped by the broader cultural context in which Chinese university students experience and manage social-emotional distress.

### 5.1 Implications

#### 5.1.1 Theoretical implications

Using ERPM and ACT as theoretical perspectives, we explore how ATMT relates to SA in university students. It focuses on emotional regulation and psychological flexibility. The findings showed that ATMT was associated with CR, ES, psychological flexibility, and SA. This model helps us understand how emotion regulation strategies and psychological flexibility are related to SA in the context of students’ ATMT. It also offers a perspective for understanding how students’ openness to music-related psychological support may be linked to anxiety-related experiences. This study also extends the application of ERPM and ACT to the study of ATMT among university students. It shows how emotion regulation strategies, psychological flexibility, and SA are related within this framework. This provides empirical support for applying these two theories to the study of university students’ social anxiety.

#### 5.1.2 Practical implications

The results provide practical guidance for mental health education related to SA among students. In university mental health education, teachers and counselors can provide students with information about evidence-based MT and music-related psychological support. They can also hold workshops on emotion regulation and psychological flexibility. These activities may be related to students’ awareness of CR, relaxation strategies, mindfulness, and acceptance-based coping. Schools can offer counseling courses or psychological support programs, especially for students with lower psychological flexibility. Such programs may be useful for helping students understand their emotional coping patterns and develop more flexible responses to social stress. Schools may also encourage students to participate in group music activities, such as choir or campus music clubs, as these activities are linked to social connection and emotional expression. However, formal MT should be delivered by trained professionals rather than general teachers. University students may also benefit from paying closer attention to self-awareness and emotional regulation in their daily lives. A more positive attitude toward MT and music-related psychological support may be associated with greater openness to emotional support, self-acceptance, and social adjustment. These measures may provide useful support for students’ mental health, but they should not be interpreted as evidence that MT directly reduces SA. Therefore, the practical implications of this study mainly concern mental health education and students’ awareness of music-related psychological support, rather than recommendations for implementing MT as a verified intervention.

### 5.2 Limitations and future research suggestions

This study shows an important connection between ATMT and SA in university students. It has some limitations. Future research can expand on and improve these areas. This study mainly used self-reported questionnaires to collect data. This method captures people’s feelings and psychological states. It can be influenced by social desirability bias and self-presentation bias. Further investigations could improve the consistency and accuracy of the results. They can use data from many sources. This could include teacher assessments, peer evaluations, or physiological indicators like heart rate and skin conductance. This would help measure changes in SA more objectively and make the results more complete. A cross-sectional design was utilized in this study. The results show the relationships between ATMT, emotional regulation, psychological flexibility, and SA. Causal connections between the variables cannot be demonstrated. In future studies, researchers may use longitudinal data to track how the relationship between ATMT and university students’ psychological status unfolds over time. The research can also look at how students adapt in different situations over time. A longitudinal design can show how SA changes over time. It can also examine its relationship with factors like emotional regulation and psychological flexibility. This would provide stronger support for causal inferences in the model. Finally, this study examined students’ ATMT rather than their participation in formal MT intervention. Thus, the results cannot support a conclusion about whether MT is effective for lowering SA. In addition, students may have differed in their understanding of MT when completing the questionnaire. Some participants may have understood MT as professionally delivered therapy, whereas others may have interpreted it more generally as music listening or music-related emotional support. Therefore, the findings should be interpreted as reflecting students’ self-reported attitudes toward MT, rather than their actual knowledge of or experience with formal MT. Further work could move beyond cross-sectional designs by testing structured MT programs delivered by qualified practitioners, with attention to both collective sessions and one-to-one formats. Future studies should provide participants with a clear definition of MT before data collection or directly assess their prior knowledge of MT. Future studies could also examine how formal MT is related to psychological flexibility training or emotion regulation skills training. Such research may provide stronger evidence for developing educational and mental health support strategies for university students.

## 6 Conclusion

This study looks at the ERPM and ACT framework. It investigates the relationship between ATMT and student’s SA. It looks at how emotional regulation acts as a mediator. It examines psychological flexibility as a moderator. The study finds a strong negative relationship between ATMT and SA. Emotional regulation, such as CR and ES, is important in this relationship. Psychological flexibility moderates it. Students with higher psychological flexibility tend to show a stronger negative association between ATMT and SA. This study creates a multivariate path model. It connects ATMT, emotional regulation, and psychological flexibility. This model helps understand factors related to SA among university students. The study confirms the link between ATMT and SA. The study also provides evidence for applying ERPM and ACT to the study of SA in this population. As SA remains an important concern for university students’ mental health, the findings highlight the value of multidimensional mental health education, including emotion regulation training, psychological flexibility support, and appropriate information about music-related psychological support. In conclusion, this study provides a theoretical basis for understanding SA among university students. It also offers practical insights for future educational practices and mental health support in university settings.

## Data Availability

The raw data supporting the conclusions of this article will be made available by the authors, without undue reservation.

## Footnotes

1
https://www.wjx.cn/
